# Fluorescence *In Situ* Hybridization and Immunohistochemistry as Diagnostic Methods for ALK Positive Non-Small Cell Lung Cancer Patients

**DOI:** 10.1371/journal.pone.0052261

**Published:** 2013-01-24

**Authors:** Pablo Martinez, Javier Hernández-Losa, M Ángeles Montero, Susana Cedrés, Josep Castellví, Alex Martinez-Marti, Natalia Tallada, Nuria Murtra-Garrell, Alejandro Navarro-Mendivill, Victor Rodriguez-Freixinos, Mercedes Canela, Santiago Ramon y Cajal, Enriqueta Felip

**Affiliations:** 1 Medical Oncology Department, Vall d'Hebron University Hospital, Universidad Autónoma de Barcelona, Barcelona, Spain; 2 Pathology Department, Vall d'Hebron University Hospital, Universidad Autónoma de Barcelona, Barcelona, Spain; 3 Thoracic Oncology Department, Vall d'Hebron University Hospital, Universidad Autónoma de Barcelona, Barcelona, Spain; Johns Hopkins University, United States of America

## Abstract

**Background:**

Anaplastic Lymphoma Kinase (ALK) positivity represents a novel molecular target in a subset of Non-Small Cell Lung Cancers (NSCLC). We explore Fluorescence *in situ* Hybridization (FISH) and Immunohistochemistry (IHC) as diagnostic methods for ALK positive patients and to describe its prevalence and outcomes in a population of NSCLC patients.

**Methods:**

NSCLC patients previously screened for Epidermal Growth Factor Receptor (EGFR) at our institution were selected. ALK positive patients were identified by FISH and the value of IHC (D5F3) was explored.

**Results:**

ninety-nine patients were identified. Median age was 61.5 years (range 35–83), all were caucasians, eighty percent were adenocarcinomas, fifty-one percent were male and thirty-eight percent were current smokers. Seven (7.1%) patients were ALK positive by FISH, thirteen (13.1%) were EGFR mutant, and 65 (65.6%) were negative/Wild Type (WT) for both ALK and EGFR. ALK positivity and EGFR mutations were mutually exclusive. ALK positive patients tend to be younger than EGFR mutated or wt patients. ALK positive patients were predominantly never smokers (71.4%) and adenocarcinoma (71.4%). ALK positive and EGFR mutant patients have a better outcome than negative/WT. All patients with ALK FISH negative tumours were negative for ALK IHC. Out of 6 patients positive for ALK FISH with more tissue available, 5 were positive for ALK IHC and 1 negative.

**Conclusions:**

ALK positive patients represent 7.1% of a population of selected NSCLC. ALK positive patients have different clinical features and a better outcome than EGFR WT and ALK negative patients. IHC is a promising method for detecting ALK positive NSCLC patients.

## Introduction

Lung cancer is the most frequent cause of cancer-related death worldwide, accounting for more than 1 million deaths per year. [1] Although cytotoxic chemotherapy remains the mainstay of treatment for the majority of patients with advanced non-small cell lung cancer (NSCLC), [2] the identification of specific genetic lesions which drive proliferation of cancer cells has led to the development of new target therapies in a subset of patients with NSCLC [3], [4]. In recent years, anaplastic lymphoma kinase (ALK) rearrangement, predominantly with the echinoderm microtubule-associated protein like 4 (EML4) gene, has been identified as an oncogenic event in a subset of NSCLC patients [4]. ALK translocation results in the constitutive expression of the tyrosine kinase domain of ALK protein, which results in tumor development and growth. The oncogenic dependence of this event is demonstrated on the basis that removal ALK kinase activity reverses the malignant pattern and growth [5]. Recently, results of a phase 1 trial evaluating an ALK inhibitor, Crizotinib, in patients with ALK positive NSCLC demonstrated encouraging results [6]. Clinical trials with Crizotinib and other ALK inhibitors in this subset population of ALK positive NSCLC patients are ongoing.

Initial reports have shown that ALK positive NSCLC patients tend to be younger, predominantly non/light smokers with an adenocarcinoma histology than the overall NSCLC patients population [7]. These clinicopathological features are also frequent in patients with EGFR mutations, but both genetic events seem to be mutually exclusive [8]. In unselected patients with NSCLC the prevalence of ALK positivity range from 1% to 7% [9], but more than 30% in patients selected for EGFR Wild-Type (WT), adenocarcinoma and no smoking history [7]. Its prevalence in a selected European population of NSCLC patients it is not yet well known.

Nevertheless, ALK positive NSCLC patients and their particular characterictics have been elucidated but a clear definition of ALK positivity remains a challenging issue. First reports on the prevalence of EML4-ALK rearrangements used RT-PCR for detecting patients, usually as a retrospective analysis of resected specimens from NSCLC patients [4], [9]. However, this method is unable to detect unknown EML4-ALK variants or rearrangements with other partners different from EML4. New platforms have been developed to supply that deficiency [10]. For selecting patients in Crizotinib trials, FISH with a break apart probe to ALK is the diagnostic method. FISH testing allows the detection of ALK translocations, no matters the partner or the variant, but ALK positivity definition by FISH and its restricted use to resected or biopsy specimens are limitations [11]. Inmunoshistochemistry (IHC) has also been explored. IHC analyses using antibodies against ALK protein used in hematologic malignances have shown poor sensitivity in patients with NSCLC, probably due to the lower ALK protein levels expressed compared to haematologic malignances with ALK rearrangements. The new high sensitivity monoclonal antibody D5F3 seems to have enough accuracy in identifying patients to be reproduced in worldwide manner [12], as all patients in which there was tissue enough in the phase 1 Crizotinib trial previously selected by FISH positivity were also positive by IHC, whereas only two of three parts of those patients were positive by RT-PCR. FISH negative samples and normal lung tissues did not express ALK protein by IHC [6]. Recently, other diagnostic methods have been also explored [13], [14].

The aim of this study is to explore the prevalence of ALK positivity in a European cohort of selected NSCLC patients by FISH, to better define its clinical features and outcomes and, to explore IHC as diagnostic method testing for ALK in NSCLC patients.

## Patients and Methods

### Patients

All included patients had received treatment or consultation from the Medical Oncology Service at Vall d'Hebron University Hospital. All NSCLC patients previously screened for EGFR mutation status between May 2006 and January 2010 were selected. EGFR mutational analyses had been performed based on a medical case per case indication, taking into account gender, histology and smoking history but without fixed parameters. Medical records were revised and basal clinicopathological features, treatments and outcomes recorded. If tissue was available for ALK analyses, patients were first tested by FISH and subsequently by IHC. The Institutional Ethics Committee reviewed and approved this study.

### Tumor samples

Unstained slides from formalin-fixed, paraffin-embedded (FFPE) tumor samples from biopsies or cell blocks reconstructed from cytology or, in the absence of this, the slides from cytology were then analyzed.

### EGFR mutational analyses

#### DNA Extraction

Samples obtained by FNA and FFPE were digested for 48 hours with proteinase K and 180 µl of G2 digestion buffer at 37^a^C, then the DNA was extracted with EZ1 DNA Tissue Kit with a Biorobot EZ1 workstation eluting the samples in 50 µl following the manufacturer's instructions. Concentration and purity of the extracted DNA were determined by spectrophotometry and then the DNA was stored at −20°C.

#### PCR Amplification and Direct Sequencing

PCR was performed in 30 µl volumes using 50 ng of template DNA, 0.75 U of AmpliTaq Gold DNA polymerase (Perkin-Elmer; Roche Molecular Systems; Branchburg, NJ), 3 µl of PCR buffer (Perkin-Elmer), 0.8 µmol/L deoxynucleotide triphosphate (Promega), 0.5 µmol/L of each primer, and concentrations of MgCl2, Exons 19 and 21 were amplified by PCR. Primer sequences were obtained as described in supplementary data. PCR program was performed by 35 cycles of denaturation at 94°C for 45 s, primer annealing at 58°C for 30 s, and elongation at 72°C for 30 s. A final extension proceeded at 72°C for 10 min. The bands of PCR products were visualized by electrophoresis in gel with BrEt. Each sample was sequenced in duplicate in both forward and reverse directions using the BigDye Terminator kit 3.1 (Applied Biosystems; Foster City, CA) and an ABI prism 310 (Applied Biosystems) according to manufacturer instructions. The sequences were then compared with the GenBank-archived human sequence for *EGFR* (accession number AY588246) by Chromas Pro Software.

#### EGFR determination by real time PCR

All cases were analyzed using the Therascreen EGFR PCR Kit (Qiagen. Manchester Ltd) following the manufacturer instructions to detect mutations in real-time PCR reactions. Real-time PCR was performed using an ABI7500Fast (Applied Biosystems; Foster City, CA) under the following conditions: Data were analyzed using 7500 software (Version 2).

### Fluorescence *in situ* Hybridization (FISH)

We prepared 4 µm paraffin-embedded histological sections for FISH analysis. The commercial Vysis LSI *ALK* Dual Color, Break Apart Rearrangement Probe (Abbott Molecular Inc., Des Plaines, IL) was used according to the manufacturer's instructions. Results were analyzed in a fluorescent microscope (Nikon 501) using the Isis Fluorescence imaging system software. A minimum of 100 nuclei was scored. A FISH positive case was defined as having more than 15% tumor cells showing separated green and red signals or single red signals identified cells with rearranged *ALK*. FISH was performed and analyzed by two different pathologists.

### Immnunohistochemistry

Briefly 3 µm-thick sections were cut from the tissue specimens and placed on poly-L-lysine–coated glass slides. All slides were stained with ALK antibody (clone D5F3 Cell Signalling Technology) diluted 1∶50 using a Ventana Ultraview DAB detection kit in a Ventana BenchMark XT processor (Ventana Medical Systems, Inc, Tucson, AZ). Antigen retrieval was a standard automated process on the Ventana BenchMark XT at 37°C for 16 minutes. All slides were analyzed by two different pathologists and classified. Samples were deemed to be IHC-positive if a tumor-specific staining of any intensity in ≥10% of the tumor cells were present.

### Statistical analyses

Unless otherwise specified, for the analyses of clinical and molecular markers on the patient samples, the Fisher's exact test was used to assess correlation between categorical variables, and Student's t test was used to assess association between the distributions of treatment outcome. All reported p values are two-sided unless otherwise specified, and we considered a test as statistically significant if p≤0.05. To compare the correlation between FISH and IHC to detect ALK positive patients we used a kappa method.

## Results

Between May 2006 and January 2010 a total of 99 patients previously screened for EGFR mutations with tissue available for ALK analyses by FISH were identified. The availability of tissue for FISH and IHC analyses was assessed as positive by the existence of a FFPE block or slides from cytology in the archives of our institution. After study procedures, there was not enough tissue to perform a valid FISH assay in 14 patients. For IHC analysis this number was higher, at 19 patients.

Basal characteristics of the patients are summarized in table 1 (table 1). Patients were predominantly adenocarcinomas (80%) and never/former smokers (62%) with equal distribution for gender.

**Table 1:** Basal characteristics.

	Years (range)
Age	61.5 (35–83)

	n (%)
Histology	
ADK	79 (80)
SCC	7 (7)
NOS	13 (13)
Sex (female)	48 (49)
Caucasian	99 (100)
Never smoker	34 (34)
Former smokers	28 (28)
Stage	
I	20
II	8
III	20
IV	51

Of the 99 tumor samples screened, 13 patients (13.1%) harbored an activating EGFR mutation, 7 patients (7.1%) were ALK positive and 65 patients (65.7%) were EGFR WT and ALK negative, and in 14 patients (14.1%) there was not enough tissue to perform a valid FISH assay for ALK (table 2). EGFR mutation and ALK positivity were mutually exclusive.

**Table 2:** Characteristics of the patients according to molecular status.

	EGFR WT/ALK−(n = 65)65.7%	EGFR mut/ALK−(n = 13)13.1%	EGFR WT/ALK+(n = 7)7.1%	EGFR WT/ALK UNK(n = 14)14.1%
Median age				
Years (range)	62.3 (36–83)	63 (36–78)	56.7 (38–78)	58.8 (35–80)
Sex				
Male (%)	36 (55.4)	3 (23)	3 (42.9)	9 (64.3)
Female (%)	29 (44.6)	10 (77)	4 (57.1)	5 (35.7)
Smoking				
Never	16 (24.6%)	9 (69.2%)	5 (71.4%)	4 (28.6%)
Former	20 (30.8%)	3 (23.1%)	-	5 (35.7%)
Current	29 (44.6%)	1 (7.7%)	2 (28.6%)	5 (35.7%)
Histology				
ADC	49 (75.4%)	13 (100%)	5 (71.4%)	12 (85.8%)
SCC	6 (9.2%)	-	-	1 (7.1%)
NOS	10 (15.4%)	-	2 (28.6%)	1 (7.1%)

**Figure 1:**
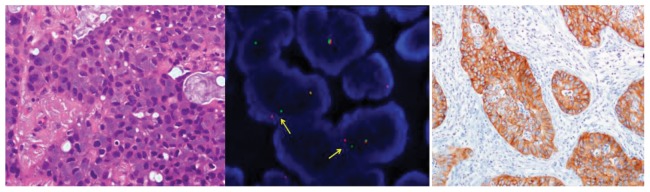
Lung adenocarcinoma with ALK rearrangement, showing a solid growth pattern with the presence of signet ring cells in some areas (HE 40×). FISH analysis using a break apart probe. Positive cells show a fusion of the red and green signals corresponding to the intact chromosome, and the split signals indicative of the ALK rearrangement (arrows). Immunohistochemistry for ALK in a NSCLC using D5F3 antibody. Tumor cell show cytoplasmic expression of the protein while the rest of cells are completely negative (20×).

ALK positive patients tend to be younger (56 years) than EGFR mutant (63 years) or EFGR WT/ALK negative (62 years) patients but these differences were not statistically different. There was not any clear gender prevalence for ALK positive patients (4 females and 3 males). Five of them were never smokers (71%) and 2 were smokers at the time of diagnosis. All ALK positive patients had a non squamous histology, 5 of 7 patients were adenocarcinoma and the other 2 patients had a Not Otherwise Specified (NOS) NSCLC. Four of the 5 ALK positive adenocarcinoma patients showed a solid and acinar growth pattern and, in addition, four of the five showed a pattern with signed ring cells was present. (Figure 1)

EGFR mutant patients had also a predominantly adenocarcinoma histology (100%) and were predominantly never smokers (71%) but with a clear gender predisposition for females (77%).

The EGFR wt/ALK negative group and the EGFR wt/ALK unknown group did not differ in their basal characteristics and were comparable to the characteristics of the entire cohort of 99 patients.

We also explored the best clinical response with an EGFR TKI or platinum based chemotherapy regimen in metastatic or relapsed patients according to EGFR and ALK status. As expected, ALK positive patients treated with erlotinib had no objective responses; however, only two ALK positive patients have been treated with EGFR TKI's. No responses for EGFR wt/ALK negative patients were seen were treated with a EGFR TKI. By contrast, a 75% of responses were seen among the group of EGFR mutant patients. Responses to first line platinum based chemotherapy were 29% for ALK positive patients, 60% for EGFR mutant patients and 40% for EGFR WT/ALK negative. (Table 3)

**Table 3:** Response to treatment (partial and complete) according to molecular status.

Treatment	ALK+	EGFRmut	EGFR WT/ALK−
Doublet chemotherapy	2/7 (29%)	3/5 (60%)	6/15 (40%)
TKI	0/2	6/8 (75%)	0/7

**Figure 2:**
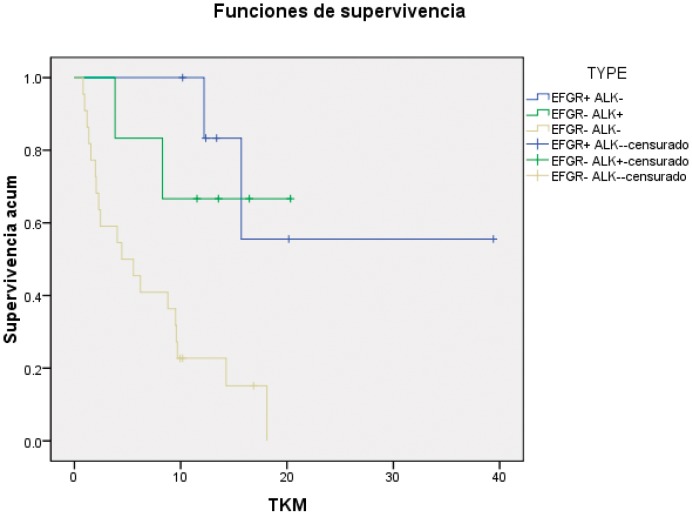
Overall survival according to molecular status.

At the time of analysis, median follow-up of the 65 patients with advanced/relapsed NSCLC was 9.5 months among the patients still alive; at that time, 57 patients had died. We analyzed overall survival (OS) of patients according to ALK and EGFR genotype. The median OS for EGFR wt/ALK negative patients were 4.5 months, for EGFR mutant patients were 15.7 months (p = 0.018) and had been not reached for ALK positive patients (p = 0.103). In the ALK positive group there were four patients (of a total of seven) that had received crizotinib as part of their treatment at sometime in the course of their disease. (Figure 2)

**Figure 3:**
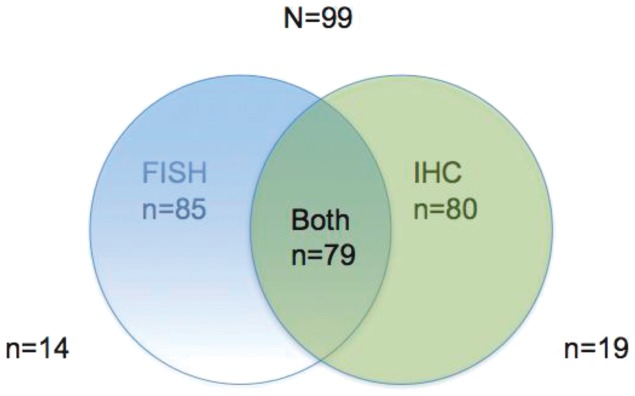
Diagram of total patients and distribution according to ALK testing result for FISH and IHC.

Finally, we performed an ALK IHC with the D5F8 antibody in the 80 patients in which there was material still available after EGFR and FISH analyses (Figure 3). All of 73 patients negative for ALK by FISH were also negative for IHC. Of six ALK FISH positive patients tested for IHC, 5 were positive and one negative. This result in a positive predictive value for IHC with D5F8 antibody of 100% and a global good agreement (Kappa 0.783, range 0.642–0.924) (Table 4).

**Table 4:** Distribution of patients results according to both ALK test methods.

	IHC			
FISH		+	−	
	+	5	1	
	−	0	73	
				79

## Discussion

ALK activation has been identified as a driver oncogenic alteration in a subset of NSCLC patients. Rearrangements involving ALK gene is an example of oncogenic dependence. ALK positive patients show predominantly an adenocarcinoma histology, never/light smoking history and younger age at diagnosis. Developement of new drugs targeting this alteration led to impressive tumor responses in this subset of patients. Recently, crizotinib, a small molecule which inhibits the tyrosin kinase activity of ALK, has been approved for treating patients with advanced ALK positive NSCLC after impressing results in early trials [6]. At the same time, a diagnostic molecular FISH test has been approved by Food and Drug Administration for detecting ALK positive patients. Taken together, these two facts, show how important is in the era of target therapies to identify and validate a biomarker for selecting those patients more suitable to achieve a benefit, even since the earliest development.

In our report, we select a subset of patients on the basis that previously determination of EGFR status let us to identify a population of patients more suitable to harbor an ALK alteration. This criteria, using a biomarker, would let us identify an enriched population of NSCLC patients in which molecular features were considered clinically relevant. In this cohort, the prevalence of EGFR mutations were 13.1%, which is close to the frequency reported by the Spanish Lung Cancer Group in a similar population [15], which indirectly indicate that our selected population represents the global amount of patients what are selecting for EGFR screening in routine practice. ALK positive patients by FISH represents in our study a 7.1% of the total, which is concordant with publications of other investigators in this population of patients [7]–[9], [16] and being the first ALK prevalence report in a cohort of predominantly metastatic European NSCLC patients.

As previously reported, ALK positive patients were predominantly adenocarcinomas, with low previous smoking history and tend to be younger than the amount of NSCLC patients. Therefore, ALK positive patients did not show responses to EGFR TKI's but a similar benefit from chemotherapy than ALK negative patients. Although previous reports have suggest that pemetrexed could be the preferred chemotherapeutic agent for ALK positive patients [17], [18], due to the small size of our ALK positive population we were not capable of perform this analyses. Interestingly, two of the ALK positive patients in our serie were current smokers at the moment of diagnosis.

Recent data seems to indicate that in absence of ALK targeted agents ALK positivity is a detrimental prognostic factor for NSCLC patients [19]. However, in patients with advanced, ALK-positive NSCLC, crizotinib therapy is associated with improved survival compared with that of crizotinib-naive controls [20]. In our study, we analized survival according to molecular status irrespective of being treated with crizotinib or other ALK inhibitor and find that those ALK positive NSCLC patients tend to live longer than EGFR wt/ALK negative patients. We did not perform an analysis of survival for ALK positive patients adjusting for crizotinib treatment due to the small size of the cohort. Nevertheless, four of the ALK positive patients had been treated with crizotinib at the moment of the survival analysis. Taken together, these results indicate that in the era of the ALK target therapies, ALK positive NSCLC patients have a better outcome than EGFR WT/ALK negative patients. However, these results should be analized with caution, one of the selection bias in this study is that patients with a worse performance status than overall lung cancer patients had been included. The main reason for that bias is that patients with better performance had been recruited into clinical trials and EGFR was not tested at our local laboratory. Another possible bias is that in patients with poor performance o greater comorbidities the EGFR mutational status was analyzed as these patients were not suitable to receive other kind of treatment than EGFR TKI's if an EGFR mutation was demonstrated. Both biases may enriched our cohort with patients with poorer outcomes and, thus, only 15 of patients EGFR wt/ALK negative patients were treated with a platinun based chemotherapy.

An important issue for clinicians is to identify those patients suitable for treatment with crizotinib. FISH analysis is the standard method. However, IHC has been explored by different groups in an attempt of identify a more worldwide suitable method for screening and diagnosis of ALK positive patients. First antibodies, previously used for diagnosis of haemathological malignances, showed have not enough sensitivity to be applied in ALK positive patients [12]. Since them, two high-sensitivity strategies have been explored. One of them is to improve test sensitivity and to develop an IHC score for selecting patients for FISH analysis [21], [22]. Another approach, is to developed high sensitivity and specificity ALK antibodies that led identify ALK positive patients by itself [23], [24]. In our study, we used a new high sensitivity and specificity monoclonal antibody D5F3 in a cohort of selected patients with NSCLC in parallel with FISH for identifying ALK positive patients. We found that all ALK positive patients by IHC were also positive by FISH. However, one FISH positive patient resulted negative by IHC. That false negative patient had no more tissue available to repeat the analysis, but had been previously recruited in a crizotinib trial with another FISH positive analysis in the central lab of the study. Taken together, we could to conclude that a positive IHC analysis with the D5F3 monoclonal antibody should be enough to select a patient for receive treatment with an ALK inhibitor as no patient positive for IHC was been found to be negative by FISH. When a clinician should consider to perform a FISH analysis in a patient with a negative result by IHC, or viceversa, is a question that should be addressed in the future. Even more, what is the meaning of a FISH test positivity in less than 15% of the cells or what should be done with those patients with no concordant results with IHC and FISH are some of the regarding issues to be standardized in the future.

To conclude, in our study we showed that the prevalence of ALK positive patients is 7.1% in a caucasian selected population of NSCLC by FISH. In the era of the ALK targeted treatments ALK positive patients have different clinical features and a better prognostic than EGFR WT and ALK negative patients. IHC with D5F3 monoclonal antibody against ALK is an accurate method for detecting ALK positive NSCLC patients.
